# GPS & GLONASS Mass-Market Receivers: Positioning Performances and Peculiarities

**DOI:** 10.3390/s141222159

**Published:** 2014-11-25

**Authors:** Paolo Dabove, Ambrogio M. Manzino

**Affiliations:** Politecnico di Torino—DIATI Department, Corso Duca degli Abruzzi 24, Turin 10129, Italy; E-Mail: ambrogio.manzino@polito.it

**Keywords:** GNSS, GLONASS, mass-market receivers, quality position, accuracy

## Abstract

Over the last twenty years, positioning with low cost Global Navigation Satellite System (GNSS) sensors have rapidly developed around the world at both a commercial and academic research level. For many years these instruments have only acquired the GPS constellation but are now able to track the Global’naja Navigacionnaja Sputnikovaja Sistema (GLONASS) constellation. This characteristic is very interesting, especially if used in hard-urban environments or in hard conditions where satellite visibility is low. The goal of this research is to investigate the contribution of the GLONASS constellation for mass-market receivers in order to analyse the performance in real time (Network Real Time Kinematic—NRTK positioning) with post-processing approaches. Under these conditions, it is possible to confirm that mass-market sensors could be a valid alternative to a more expensive receiver for a large number of surveying applications, but with low cost hardware the contribution of the GLONASS constellation for fixing ambiguities is useless, if not dangerous.

## 1. Introduction

Currently, the use of GNSS positioning is a common practice because GPS/GNSS receivers are included in various instruments, such as smartphones and cars. Many types of receivers and antennas are available, depending on cost and application. Receivers range from geodetic triple frequency and multi-constellation (which currently cost over 5000 euros) to mass-market instruments that typically use only the GPS constellation and pseudorange measurements of at least the L1 (single frequency) GPS frequency, which costs a few hundred euros. Besides the cost, these receivers differ in positioning accuracy and precision; the first one allows us to obtain centimeter accuracy in real-time positioning (using Continuous Operating Reference Stations—CORSs) and a sub-centimeter accuracy in post-processing [1], after a network adjustment.

The second class of receivers are (in theory) less precise compared to the first type and allow us to obtain an accuracy of a few meters in real-time stand-alone positioning and an handful of centimeters (*i.e.*, four to six cm) in real-time if a CORSs network is considered [2]. Regarding post-processing, it is possible to obtain an accuracy of about two to five cm, if considering a Virtual Rinex: this is a data file of a virtual station generated by the network software that manages the CORSs network [3].

The use of mass-market receivers is widespread, primarily thanks to their low cost. GPS chips are produced in millions of copies and costs only a few euros. Very often these receivers are assembled in ‘evaluation kits’ (composed of a receiver and a patch antenna) with a cost of about €200–300 and more often they are coupled with inertial sensors [4–6]. All these instruments are able to track the GPS signals, but a few of them are also able to track the GLONASS constellation [7,8]. The goals of this research are to analyse and test the performance of this last type of receiver in order to understand whether a multi-constellation receiver is useful for mass-market applications (*i.e.*, mobile mapping, navigation, precise farming, *etc.*).

Some of them are also able to manage differential corrections broadcasted by software that manages a CORSs network while others are only able to make a stand-alone positioning. In the first case, with this type of receiver, it is also possible to do a Network Real-Time Kinematic (NRTK) positioning [9]. Some are also able to store raw data (pseudorange, carrier-phase, and Doppler measurements) in their internal memory, while others need an external device to store data (*i.e.*, a laptop), and still others are not able to save any raw data.

Some tests were designed at the Politecnico di Torino in order to demonstrate the performance of mass-market receivers for various purposes (mobile mapping [10], precise farming, disaster management [11], surveying and mapping [12]) considering receivers able to track only the GPS constellation; the goal of this work is to analyse the performances of mass-market receivers that are able to track the GLONASS constellation [8,13,14] in order to demonstrate the benefits of multi-constellation receivers for mass-market instruments.

## 2. Experimental Section

In order to test the benefits of the GLONASS constellation using mass-market receivers, two different types of receivers were considered: a single constellation (GPS) single frequency L1 receiver and a double constellation single frequency receiver. The characteristics of these instruments are shown in Table 1.

At the same time, previous studies [2,6] have demonstrated the importance of the GNSS antenna, especially when mass-market receivers were used. For this, we considered two different antennas: the first one is a geodetic and multi-constellation antenna (LEIAR10 by Leica Geosystems^®^, Heerbrugg, Canton St. Gallen, Switzerland) and the second one is a mass-market antenna provided by the Garmin^®^ Company (Schaffhausen, Switzerland). The characteristics of these antennas are shown in Table 2. Antennas coupled to mass-market receivers (patch antennas) typically have a higher gain because they must receive signals in hostile environments and indoor positioning [16].

In these cases we must choose a compromise between cost and performance, such as a Garmin GA38 antenna. We decided to divide the performance analysis of these types of receivers into two subsections: the first is dedicated to the post-processing approach and the second focuses on real-time kinematic positioning. For these two sub-sections, a CORSs network was considered. This infrastructure is composed of various master stations that include geodetic (multi-frequency and multi-constellation) receivers.

### 2.1. Post-Processing Approach

Raw data acquired in a GNSS static survey can be post-processed with commercial or scientific software, using both real data obtained from CORSs and synthetic data (Virtual Rinex, hereinafter VR) created by the network software. Great importance is given to the distance between master and rover [1,6,17]; some studies show the behaviour of mass-market receivers in real time and in post-processing positioning.

The correct fixing of the phase ambiguity for single-frequency receivers has been studied for many years, [18,19] and for this reason the master-rover distances are usually limited to five to ten km. In mass-market receivers, the problem is even more complex, due to the noise of the code and carrier phase data [6].

For these reasons, different tests were made for the function of master-rover distance: we compared the solutions obtained by NVS and u-blox receivers when a master station is five, ten, and 25 km from the rover for a session length of five, ten, and 15 min. At the same time, the session length plays an important role in post-processing performances; it is important to know the time that the user must wait before completing a survey in order to have a fixed ambiguity phase on an L1 frequency to obtain centimetric accuracy.

**Figure 1.**
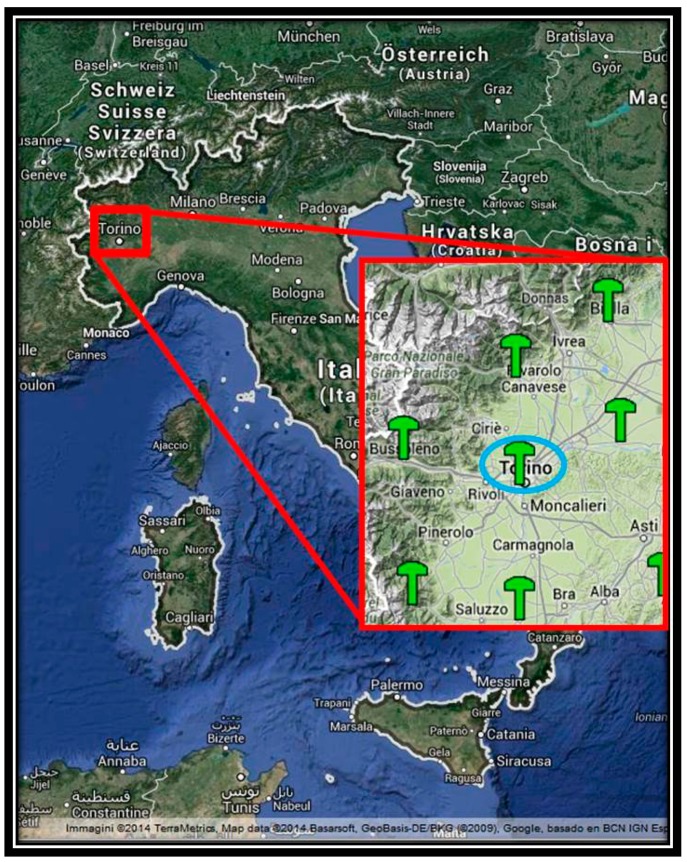
GNSS Regione Piemonte GBAS NRTK Network [20].

The data files were processed by the commercial software Leica Geomatics Office™ v.8.3 by Leica Geosystems^®^, which is based on the double differences approach, and for generating the VR, we used Leica Spidernet^®^ as a network software: the network configuration is shown in Figure 1.

### 2.2. Real-Time Approach

A well-known and more accurate real-time positioning technique is the Real Time Kinematic (RTK) technique, where two geodetic receivers are used, and for limited inter-station distances (around ten to 15 km between master and rover), it is possible to determine the ambiguity value as an integer number in RT (Real Time) in order to obtain centimetric accuracy.

The Ground Base Augmentation System (GBAS) positioning that uses a network of permanent stations has allowed the breakdown of this limitation and the improvement of the reliability of positioning. With the use of geodetic receivers, the differential corrections provided by these networks allow Network RTK (NRTK) positioning.

These infrastructures are managed by control centres, which manage and broadcast differential corrections [18] to the rover: these corrections are composed of spatial correlated biases of the GNSS signal (such as ionospheric and tropospheric corrections, *etc.*) that can be broadcast to the rover in various ways (Virtual Reference Station^®^—VRS, Master Auxiliary Concept—MAC, Flächen-Korrektur-Parameter—FKP, and Nearest correction–NRT) due to the Radio Technical Commission for Maritime Services (RTCM) protocol [21] that are well described in literature [22] in order to fix the ambiguity on the carrier phase measurements [23,24].

Permanent stations usually have inter-distances of about 60 km. These infrastructures are not designed for use with mass-market receivers; however, the VRS^®^ correction may provide a useful improvement of the positioning accuracies of these receivers and with some limitations (the low accuracy and the time to fix) we can achieve the phase ambiguity fixing. Later we will demonstrate the limits and the results obtained with the two mass-market receivers.

**Figure 2.**
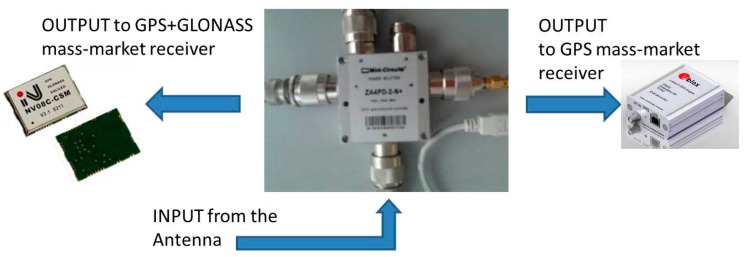
Using the antenna splitter, the positioning results of two receivers can be properly compared.

Also for these real-time experiments, we considered the NRTK Regione Piemonte network shown in Figure 1. The figure shows the area where the measurements were carried out. This network has a typical Italian mean inter-station distance (about 60 km) and can be used as a representative network in order to evaluate the performances of the receivers previously cited. In order to evaluate the performances of the instruments described in Table 1, we decided to compare the positioning performed with the u-blox and NVS receivers at the same time. To do this, a splitter was used: this instrument allows splitting the GNSS signal, which arrives at the antenna for more than one receiver (Figure 2).

**Figure 3.**
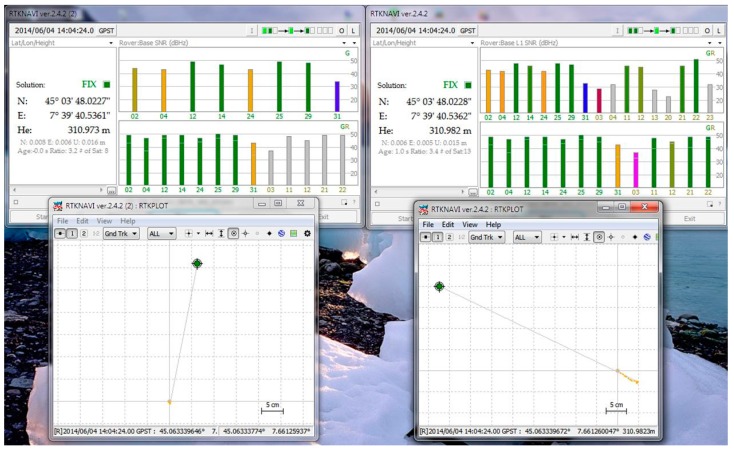
RTNAVI interface: parallel analysis of u-blox and NVS positioning.

To perform NRTK positioning, the routine RTKLIB V. 2.4.2 was used [25]. In particular for these experiments the RTKNAVI tool was used. This tool allows input for both raw data (pseudorange and carrier-phase measurements) of the u-blox and NVS receivers (simultaneously, as in Figure 3) and stream data coming from a network with NTRIP authentication. The RTKNAVI software uses a modified LAMBDA method for fixing integer ambiguities. The LAMBDA method [26] has been widely used in GNSS positioning. For real time applications computational speed is crucial: the modified LAMBDA (MLAMBDA) method [27] reduces computational complexity of the LAMBDA method.

Considering these experiments, we have fixed the ambiguities using a “ratio” equal to 3 both for the GPS and GLONASS constellations using a “continuous” method. This means that the software verifies whether the fix of the phase ambiguity is correct at each epoch. Moreover, we have chosen a cut-off angle of 10° in order to neglect the signals coming from satellites with low elevations (that are very noisy).

## 3. Results and Discussion

In the following sections we will show the results and analyse post-processing and real-time performances separately.

### 3.1. Post-Processing Approach

As previously stated, the first tests were made with consideration for single baseline positioning that was obtained with a CORS and various VR (changing the inter-station distance between virtual stations and rover along with different session lengths) in a post-processing approach. Some interesting results were obtained that are described as follows:

The first experiment corresponded to the CORS post-processing approach: we used a real permanent station eight km from the rover. Data were acquired simultaneously using the same antenna (Garmin GA38) connected to the splitter using a sampling rate of 1 s. The average values of Position Diluition of Precision (PDOP) were approximately 1.2. As stated, the results were obtained in the “single base” mode using commercial software (Leica Geomatics Office™ v.8.3 by Leica Geosystems^®^).

As seen in Table 3, both NVS and u-blox receivers can fix the phase ambiguity of a session length equal to five minutes while for a longer session (ten minutes) only the double constellation receiver was able to fix the phase ambiguity. Analysing the results, a strange behaviour in the last receiver is observed. In this last case (session length of five minutes) despite the phase ambiguity being fixed, it is noted that the results are incorrect: the planimetric coordinates are approximately 66 cm from the “true” coordinates (where “true” means the adjusted coordinates obtained with the Bernese 5.0 software in the post-processing approach using two geodetic receivers) while the altimetry difference is about 1.6 m! On the other hand, the u-blox receiver is not able to fix the phase ambiguity but provides better results. The difference between the “true” and estimated coordinates is about 9 cm using the planimetric components and only two cm for the altimetric ones. The results in terms of the differences between “true” and estimated coordinates are shown in Table 2.

It should be noted that with the NVS receiver and five minutes of data, the software declares to have reached ambiguity fixing, but only for GPS measurements. These ambiguity values are clearly incorrect. We have also performed these type of tests in different days (12 sessions in 5 days), considering also different hours (night and day) in order to analyse the results also in terms of number of visible satellites and atmospheric conditions: in Table 3 are shown the main statistical parameters that represent the obtained results. We have computed the accuracy considering the 95% of significance level, in order to have a complete analysis from a statistical point of view.

If we consider the NVS receiver, comparing Tables 3 (top) and 4 no substantial differences can be found except for the fixing of the phase ambiguity that is not reached. Despite this, in these experiments the accuracy and precisions are comparable both for the u-blox and the double constellation receiver.

Analyzing Tables 3 and 5 (that represent the same experiments with two different antennas), it is possible to see the importance of the antenna: the geodetic one decreases the noise of results for both types of receivers and it excludes the presence of a false fix, that is present in the double constellation receiver (Table 5 (top)), even if the ambiguity fixing is not reached considering a session length of 5 min. If we compare Tables 4 and 6 it is possible to underline again the importance of the geodetic antenna, that is able to increase both the accuracy and the precision of the NVS receiver, even if the GPS constellation is considered.

Thus it is possible to affirm that the GLONASS constellation is not useful for this type of receivers when using the post-processing approach if a maximum session length of ten minutes and a real permanent station are considered.

The next step is to analyse the performances of these two receivers in a CORSs network using the VR: we have decided to create a VR 5, 10, and 25 km from the rover to simulate the typical and maximum distance of VR positioning, respectively. Session lengths of five, ten, and 15 min were considered for these experiments.

As shown in Table 7, considering a session length of five minutes, neither NVS nor u-blox receivers were able to fix the phase ambiguity; despite that, only the float solutions of u-blox receiver were quite good; considering the NVS receiver the difference between “true” and estimated coordinates are approximately 30 cm for each planimetric component and 50 cm for the height, while for u-blox receivers the performances obtained considering a VR five km from the rover are very good: the differences are about five cm for planimetric components and 28 cm for the height. If the distance between VR and u-blox increases, the performances were reduced and the planimetric differences were about 20 cm, even if the altimetric one was the same. The results for the differences between estimated and reference positioning are shown in Table 7. Comparing these results with Table 3, we can affirm that a session length equal to 5 min is not sufficient to fix the phase ambiguity of both receivers: despite this, no false fix can be found. It is possible to obtain decimetric accuracies even if the σ has values from 2 up to 5 cm.

If the session length increases up to ten minutes, the performances improve and the gap between estimated and reference positions decrease; while the NVS receiver differences are greater (the only improvement is on the height), considering the u-blox receiver very good results were obtained for planimetric components. It is strange that when considering the NVS receiver and the VR five km far from the rover, the software is able to fix the phase ambiguity but provides the wrong coordinates. In fact we have differences of about 25 and 49 cm for planimetric and altimetric components respectively, while the differences are 7 and 11 cm considering the u-blox. In this case it is possible to define the NVS position as a false fix (because the differences of “true” and estimated positions are greater than the L1 wavelength), which means that the software incorrectly fixes the phase ambiguity.

Analysing the results obtained with a real CORS and the VR 8 and 10 km far from the rover respectively, some differences can be found. These differences were due to the feasibility of the VR: considering the NVS receiver and comparing Table 7 (top) (only for a baseline of about 10 km) with Table 3 (top) it is possible to note that better results (in terms of precision and accuracy) can be obtained with the VR. Comparing Table 8 (top) with Table 3 (top) (always for a baseline of about 10 km) the permanent station provides better results and the software for post-processing is able to fix the phase ambiguity in a correct way (Table 3 (top), 2nd row).

Considering the u-blox receiver for a session length of 5 min (Tables 3 (bottom) and 7 (bottom)) and a session length of 10 min (Tables 3 (bottom) and 8 (bottom)) and the VR 10 km far from the rover, the best results (both in terms of precision and accuracy) are obtained with the CORS. So in general it is possible to affirm that in this case this network product (VR 10 km far from the rover) is not useful for this class of receivers with this type of mass-market antenna if a real CORS is available with comparable inter-station distances.

Considering the results obtained with the Garmin antenna and the VR for a session length of 5 min (Table 7) and 10 min (Table 8), no substantial differences can be found with exception of the fix of the phase ambiguity for the shortest baseline (5 km): in this case it is possible to note the false fix obtained with the double constellation receiver, as it is just seen in Table 3. If the session length increases up to 15 min, no significant improvements can be found in the results. As it is just affirmed, the software used for data post-processing is able to manage better the only GPS data (both of u-blox and NVS) than the GPS + GLONASS ones (comparison of Tables 9 and 10) probably because the GLONASS data are more noisier than the GPS ones.

Focusing the attention on the type of antenna, comparing Table 9 (top) with Table 11 (top) we can affirm that the double frequency and double constellation antenna slightly improve the results considering GPS + GLONASS data while not improvement can be found if the only GPS constellation is considered. Comparing Tables 11 and 12, we can affirm that the GLONASS data are more noisier than the GPS ones even if a geodetic antenna is considered.

If the session length increases to up to 15 min more, no better results can be obtained. The NVS receiver gives better results with a VR that is 25 km from the rover than when a VR is five km away. In this case the ambiguity phase is defined as a float, while in the first case the ambiguity is fixed. Considering the u-blox receiver, the results seem reasonable; with a VR five km from the rover we obtain a fixed positioning, while if this distance increases, the solution is defined as a float. As shown in Table 9, no better results can be obtained with respect to a session length equal to 10 min.

In general it is possible to affirm that the GLONASS constellation it is not useful for these types of receivers even if VR are considered from a post-processing approach. It must be stated that these results are obtained considering the same satellite constellation and the same observations; this is possible to do by splitting the signal that comes from the antenna into two receivers (Figure 2).

Finally, Tables 11 (bottom) and 12 show the results obtained with 15 min of GPS measurements with the Leica LEIAR10 antenna. The results are better than those obtained with the Garmin antenna; however, they are very similar between the two receivers. Even the positions declared “float” have accuracies comparable to those declared “fix”. It must be underlined again that we have summarized in the previous tables the results obtained considering 12 sessions in 5 days. We have decided to show mean values and the obtained accuracy considering the 95% of significance level, in order to have a complete analysis from a statistical point of view.

### 3.2. Real-Time Approach

First of all, we decided to compare the solutions obtained by these two receivers considering the geodetic antenna: this choice was made in order to establish which is the best accuracy available with these types of receivers. In this case we have considered only the VRS correction.

In Table 13, we show the performances of NVS and u-blox receivers in a NRTK positioning mode. In order to make more clearer the results, remember that in the NMEA message:

‐ DGPS = 1 ➔ stand-alone positioning
‐ DGPS = 2 ➔ DGPS positioning
‐ DGPS = 4 ➔ RTK positioning with FIX phase ambiguity
‐ DGPS = 5 ➔ RTK positioning with FLOAT phase ambiguity

**Figure 4.**
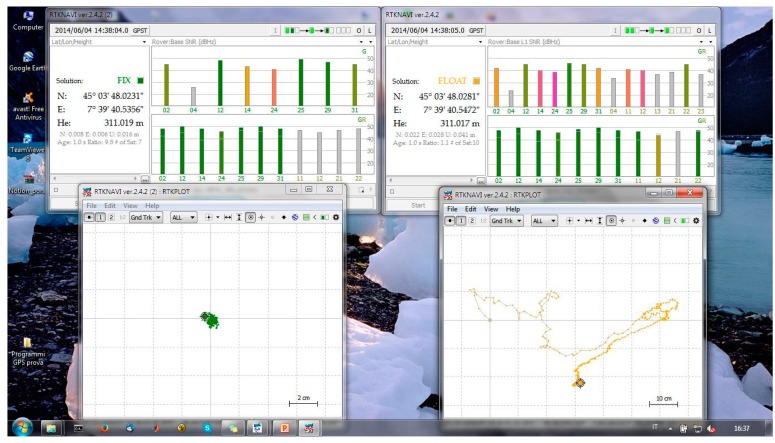
Float positions obtained by the NVS receiver (RTKNAVI window).

Despite the similar results in terms of accuracy (Tables 13 and 14), the u-blox receiver allows us to fix the ambiguity phase twice with respect to the NVS receiver considering the VRS correction and a geodetic antenna. If a low-cost antenna is considered (Table 15) the u-blox receiver is able to fix the ambiguity phase at 97% of the epochs and because the NVS cannot fix any epoch, 100% of the epochs are defined as a float. As shown in Figure 4, the float solutions are not good solutions because they are about 50 cm from the reference position. In order to verify whether it is the GLONASS constellation is the problem (in the sense that the software has problems fixing the ambiguity phase of GLONASS satellites), some tests are designed to consider only the GPS satellites.

Analysing Tables 14 and 15, it is possible to observe that the geodetic antenna improves the u-blox NRTK results considerably, but not the NVS ones if we use this as a double constellation receiver. In fact, in both cases, no fixed epochs are reached with this last receiver. If this receiver is used in a single constellation mode, a small improvement can be obtained but, in spite of all, the u-blox results are the best that it can be obtained (Table 16). In general it is possible to affirm that the best choice is to use only the GPS constellation also for NRTK positioning.

**Figure 5.**
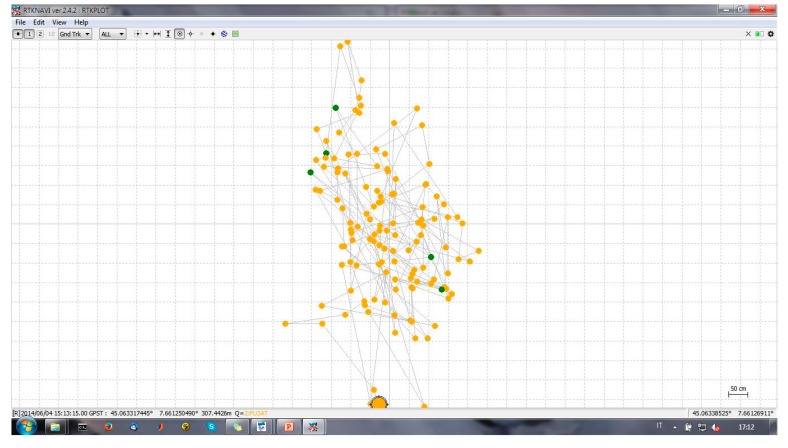
Fixed (in green) and float (in yellow) positions obtained with NVS receiver and Garmin antenna, considering the VRS correction (RTKNAVI Window).

Also considering a low cost antenna and only the GPS constellation (Table 17) we have obtained unsatisfactory results; the fixing percentage is very low (less than 8%) and the quality of float positions is very bad. As shown in Figure 5, the point cloud is very large so the solutions are more than five meters from the correct position. Also the epochs with fixed phase ambiguity (in green in the previous figure) are very far from each other. This means that we are in the presence of several false fixes of the ambiguity phase, which are very dangerous conditions for positioning. At the same time, the u-blox receiver provides better results both in terms of fixing percentage and in the quality of the positioning.

### 3.3. RTK Approach—Dynamic Tests

In order to have a more complete analysis about the performances of these types of receivers and antennas, also some dynamic tests were made. All experiments were performed by mounting the antennas on a mobile vehicle, using an antenna-splitter which allowed the simultaneous GNSS reception for three receivers (the two mass-market units shown in Table 1 and the Leica Geosystems^®^ Leica GX1230 geodetic receiver used only to verify the accuracy of the performed trajectories).

To understand if there might be some benefits in the use of different antennas, some experiments in NRTK modality were carried out, driving complex trajectories in a parking of a shopping center, always considering the VRS stream of the Regione Piemonte CORSs network.

**Figure 6.**
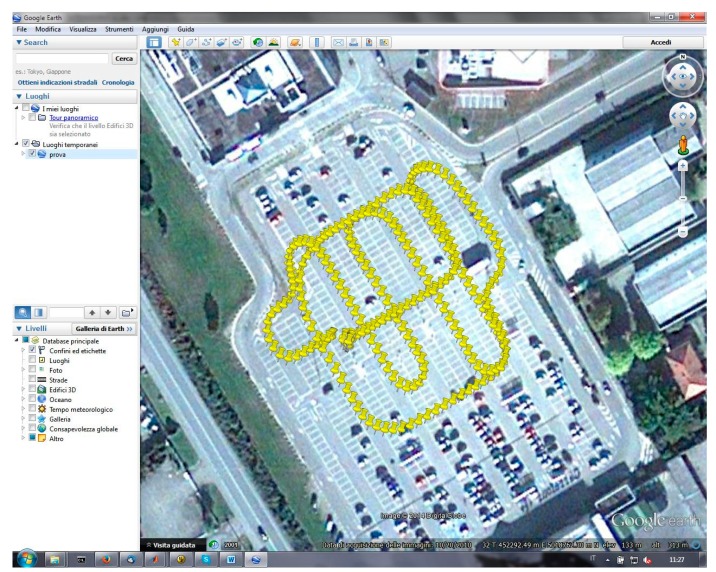
Trajectory performed in NRTK mode.

Four different tests were performed, considering the same trajectory, shown in Figure 6:

(a) u-blox, NVS and Leica receiver with the LEIAR 10 antenna, considering GPS + GLONASS constellations
(b) u-blox, NVS and Leica receiver with the LEIAR 10 antenna, considering only the GPS constellation
(c) u-blox, NVS and Leica receiver with the Garmin antenna, considering GPS + GLONASS constellations
(d) u-blox, NVS and Leica receiver with the Garmin antenna, considering only the GPS constellation

In order to make the obtained results more easy to analyse, the following tables resume some statistical parameters evaluated considering the difference between the estimated (by the mass-market receivers) and the reference (by geodetic receiver) positions.

Analysing the results with the geodetic antenna (Table 18), no epochs with fixed phase ambiguity are obtained if the NVS receiver is used. At the same time, the u-blox receiver is able to fix the phase ambiguity in 85% of epochs with a high level of precision (less than 2 cm in planimetry) and accuracy (about 5 cm in planimetry and 7 in height). Using the same antenna, the results change if the only GPS constellation is considered (Table 19): also in this case no substantial differences (in terms of precision, accuracy and percentage of epochs with fixed phase ambiguity) are obtained with the single constellation receiver while the NVS is able to fix the phase ambiguity in 72% of considered epochs, even if both precision and accuracy are less than those obtained with the u-blox.

The same results can be obtained if the Garmin antenna is used (Table 20): considering the NVS receiver, it is possible to obtain some epochs with fixed phase ambiguity only if the GPS constellation is used (Table 21); despite that, the precision and accuracy of NVS receiver is less than the u-blox one, also in this case. In this context, we must to take into account the different modeling of the clock errors in the GPS and GLONASS satellites. This means that the processing software can model these errors in a less accurate way if single-frequency receivers are considered in real-time.

Therefore, it is easier to obtain a good fixing of the phase ambiguity with a receiver that uses only a Code Division Multiple Access (CDMA) constellation with respect to one that uses a mixed CDMA and Frequency Division Multiple Access (FDMA) constellations, especially if single-frequency receivers are considered

## 4. Conclusions

The development of GNSS mass-market receivers is very fast, and new technologies evolve continuously. In this context some GNSS mass-market receivers that also consider the GLONASS constellation were developed in these last few months. In this contribution, the authors decided to analyse the benefits of this constellation with respect to the results available with a receiver able to track only the GPS constellation. Different positioning methods were considered in order to understand the performances both in real-time and in post-processing, and the obtained results are quite strange.

Considering the post-processing approach, we designed tests installing both a mass-market and a geodetic antenna on a pillar with well-known coordinates (obtained with a geodetic receiver after an adjustment with Bernese 5.0 software); different sessions lengths and inter-station distances between master (real CORS or Virtual Rinex) were considered in order to generalize the results as best as possible.

Considering a CORS station as master, the behaviour of the two receivers is not identical; the results are quite the same if a session length of ten minutes is considered; however, when decreasing the time of the survey (5 min), the results became very different: the single constellation receiver continued to provide the same results in terms of accuracy while with the NVS receiver no reasonable results were obtained. If a Virtual Rinex is considered as master station, we obtain no improvement in terms of accuracy; in all cases the u-blox receiver allowed us to obtain the best results.

Considering the real-time approach, if a geodetic antenna is considered, the accuracy obtained with two receivers with VRS correction is the same, but the fixing percentage is different. With the u-blox receiver, about 67% of epochs can be declared as fixed *versus* only 33% with the NVS. If the Garmin^®^ antenna is considered, no result comparison is possible with the VRS correction because the NVS receiver is not able to fix any epochs: in fact all epochs are defined as float solutions and the planimetric dispersion is about 80 cm at 95%. In order to verify if the GLONASS constellation can be dangerous for the real-time positioning with this type of receiver, the authors decided to repeat the same experiments excluding the Russian constellation. In this case, the NVS receiver is able to fix the ambiguity phase in 8% of cases with respect to 97% of the u-blox. We also demonstrate the quality of the positioning when the ambiguity can be declared as fixed; we have shown that the presence of a false fix is frequent, so it is possible to affirm that the VRS correction is not a good product for these types of instruments. If a single constellation is considered, we noted an improvement in results that, despite all, does not entail the use of these instruments.

Analysing the results obtained with a geodetic antenna respect to the Garmin one, no substantial differences in terms of accuracy can be found for both receivers. It is not the same if we consider the noise of results: nevertheless it is not possible to reach the fixing of the GLONASS phase ambiguity even if a geodetic antenna is considered both for real-time and post-processing approaches.

## Figures and Tables

**Table 1.** Characteristics of receivers.

**Receivers**		
	**LEA EVK-5T (U-Blox)**	**NVS NV08-CSM (Leica Geosystems)**
**Image**	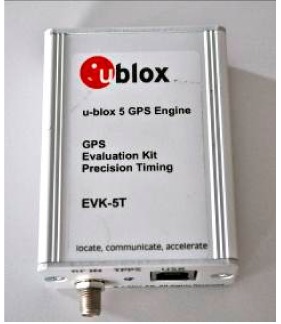	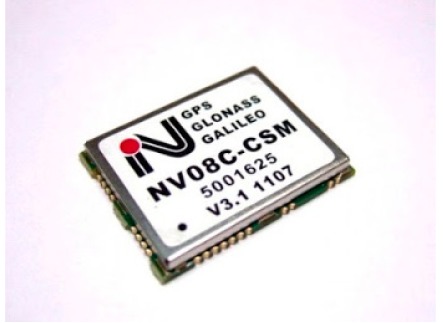
**Default Antenna**	Patch	patch
**Nr. of channels**	50	32
**Constellations**	GPS	GPS + GLONASS + GALILEO
**Type of observations**	GPS: C/A, L1, Doppler, S/N	GPS: C/A, L1, Doppler, S/N GLONASS: L1 Galileo:E1
**Position update rate**	0.25/1000 Hz	1/10 Hz
**Corrections type**	RTCM 2.x, RTCM 3.0, SBAS (WAAS/EGNOS/MSAS/GAGAN) AssistNow Online & Offline [15]	RTCM 2.x, RTCM 3.0, SBAS (WAAS/EGNOS/MSAS/GAGAN)
**Raw data format**	Ubx	BINR/BINR2

**Table 2.** Antennas considered.

**Antenna Type**	**Garmin GA38**	**LEIAR10 (Leica Geosystems^®^** **)**
**Image**	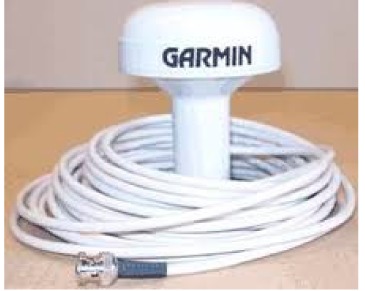	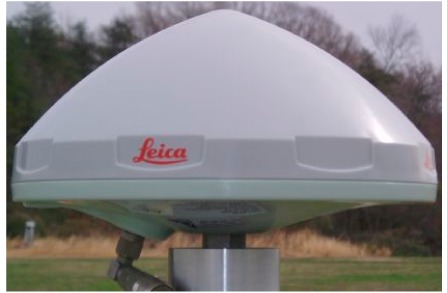
**Frequencies and constellations**	single frequency (L1) GPS + GLONASS	double frequencies (L1 + L2) GPS + GLONASS
**Gain**	27 dB on the average	About 17 dB

**Table 3.** Differences between “true” and estimated coordinates of NVS (**Top**) and u-blox (**Bottom**) receivers using a CORS eight km from the rover.

	**NVS (Garmin Antenna, GPS + GLONASS)**						

	**μE** **[m]**	**μN [m]**	**μh [m]**	**σE [m]**	**σN [m]**	**σh [m]**	
5 min	0.004	0.655	−1.599	0.012	0.019	0.026	FIX GPS
10 min	−0.011	0.015	0.029	0.008	0.011	0.019	FIX GPS

	**u-blox (Garmin Antenna, only GPS)**						

	**μE [m]**	**μN [m]**	**μh [m]**	**σE [m]**	**σN [m]**	**σh [m]**	

5 min	0.002	0.006	0.020	0.003	0.002	0.008	FIX
10 min	−0.084	−0.050	0.022	0.011	0.018	0.012	FLT

**Table 4.** Differences between “true” and estimated coordinates of NVS receiver using a CORS eight km from the rover—only GPS constellation.

	**NVS (Garmin Antenna, only GPS)**						

	**μE [m]**	**μN [m]**	**μh [m]**	**σE [m]**	**σN [m]**	**σh [m]**	
5 min	−0.008	0.012	0.045	0.010	0.032	0.019	FLT
10 min	0.009	0.010	0.030	0.003	0.002	0.010	FIX

**Table 5.** Differences between “true” and estimated coordinates of NVS (**Top**) and u-blox (**Bottom**) receivers using a CORS eight km from the rover with GPS + GLONASS constellation and LEIAR10 antenna.

	**NVS (LEIAR10 Antenna, GPS + GLONASS)**						

	**μE [m]**	**μN [m]**	**μh [m]**	**σE [m]**	**σN [m]**	**σh [m]**	
5 min	0.011	0.090	0.046	0.018	0.043	0.021	FLT
10 min	0.004	0.005	0.020	0.002	0.003	0.005	FIX GPS

	**u-blox (LEIAR10 Antenna, only GPS)**						

	**μE [m]**	**μN [m]**	**μh [m]**	**σE [m]**	**σN [m]**	**σh [m]**	

5 min	0.003	0.008	0.019	0.002	0.002	0.005	FIX
10 min	0.002	0.006	0.013	0.002	0.002	0.004	FIX

**Table 6.** Differences between “true” and estimated coordinates of NVS receiver using a CORS eight km from the rover with only GPS constellation and LEIAR10 antenna.

	**NVS (LEIAR10 Antenna, only GPS)**						

	**μE [m]**	**μN [m]**	**μh [m]**	**σE [m]**	**σN [m]**	**σh [m]**	
5 min	−0.003	0.018	0.036	0.005	0.006	0.009	FIX
10 min	−0.001	0.006	0.019	0.002	0.003	0.005	FIX

**Table 7.** Difference between estimated and reference positioning of NVS (**Top**) and u-blox (**Bottom**) receivers using a VR at various distances for a session length of five minutes.

	**NVS (Garmin Antenna, GPS + GLONASS, Session Length = 5 min)**						

	**μE [m]**	**μN [m]**	**μh [m]**	**σE [m]**	**σN [m]**	**σh [m]**	
5 km	0.309	0.294	−0.559	0.038	0.022	0.046	FLT
10 km	0.303	0.299	−0.525	0.034	0.026	0.051	FLT
25 km	0.259	0.279	−0.463	0.036	0.029	0.050	FLT

	**u-blox (Garmin Antenna, only GPS, Session Length = 5 min)**						

	**μE [m]**	**μN [m]**	**μh [m]**	**σE [m]**	**σN [m]**	**σh [m]**	

5 km	0.048	0.018	0.283	0.012	0.009	0.034	FLT
10 km	0.162	0.041	0.264	0.026	0.018	0.035	FLT
25 km	0.204	0.018	0.298	0.030	0.012	0.033	FLT

**Table 8.** Difference between estimated and reference positioning of NVS (**Top**) and u-blox (**Bottom**) receivers using a VR at various distances for a session length of 10 min.

	**NVS (Garmin Antenna, GPS + GLONASS, Session Length = 10 min)**						

	**μE [m]**	**μN [m]**	**μh [m]**	**σE [m]**	**σN [m]**	**σh [m]**	
5 km	0.273	0.263	−0.493	0.003	0.002	0.009	FIX
10 km	0.270	0.268	−0.460	0.011	0.016	0.049	FLT
25 km	0.197	0.221	−0.355	0.029	0.031	0.044	FLT

	**u-blox (Garmin Antenna, only GPS, Session Length = 10 min)**						

	**μE [m]**	**μN [m]**	**μh [m]**	**σE [m]**	**σN [m]**	**σh [m]**	

5 km	0.073	−0.018	0.108	0.002	0.003	0.005	FIX
10 km	−0.023	0.065	0.232	0.008	0.012	0.013	FLT
25 km	−0.194	−0.093	0.556	0.018	0.012	0.032	FLT

**Table 9.** Difference between estimated and reference positioning of NVS (**Top**) and u-blox (**Bottom**) receivers using a VR at various distances for a session length of 15 min.

	**NVS (Garmin Antenna, GPS** **+** **GLONASS, Session Length** **=** **15 min)**						

	**μE [m]**	**μN [m]**	**μh [m]**	**σE [m]**	**σN [m]**	**σh [m]**	
5 km	0.268	0.213	−0.386	0.032	0.019	0.033	FLT
10 km	0.338	0.264	−0.429	0.041	0.022	0.038	FLT
25 km	0.053	−0.045	0.061	0.002	0.002	0.004	FIX

	**u-blox (Garmin Antenna, only GPS, Session Length** **=** **15 min)**						

	**μE [m]**	**μN [m]**	**μh [m]**	**σE [m]**	**σN [m]**	**σh [m]**	

5 km	0.081	−0.026	0.124	0.002	0.001	0.003	FIX
10 km	0.275	−0.095	0.049	0.019	0.017	0.021	FLT
25 km	0.295	0.091	0.129	0.026	0.024	0.031	FLT

**Table 10.** Difference between estimated and reference positioning of NVS receiver using a VR at various distances for a session length of 15 min—only GPS constellation.

	**NVS (Garmin Antenna, only GPS, Session Length** **=** **15 min)**						

	**μE [m]**	**μN [m]**	**μh [m]**	**σE [m]**	**σN [m]**	**σh [m]**	
5 km	0.080	0.032	−0.091	0.003	0.002	0.008	FIX
10 km	0.092	0.048	−0.114	0.003	0.004	0.010	FIX
25 km	0.169	−0.085	0.081	0.018	0.011	0.021	FLT

**Table 11.** Difference between estimated and reference positioning of NVS (**Top**) and u-blox (**Bottom**) receivers using a VR at various distances for a session length of 15 min, considering the LEIAR10 antenna splitted, and GPS + GLONASS constellation.

	**NVS (LEIAR10 Antenna, GPS** **+** **GLONASS, Session Length** **=** **15 min)**						

	**μE [m]**	**μN [m]**	**μh [m]**	**σE [m]**	**σN [m]**	**σh [m]**	
5 km	0.092	−0.008	−0.124	0.005	0.003	0.008	FIX
10 km	0.237	0.162	0.065	0.023	0.020	0.021	FLT
25 km	0.173	0.112	0.187	0.025	0.023	0.038	FLT

	**u-blox (LEIAR10 Antenna, only GPS, Session Length** **=** **15 min)**						

	**μE [m]**	**μN [m]**	**μh [m]**	**σE [m]**	**σN [m]**	**σh [m]**	

5 km	0.075	−0.013	0.100	0.004	0.002	0.009	FIX
10 km	0.255	0.141	−0.040	0.021	0.016	0.011	FLT
25 km	0.143	0.080	0.138	0.022	0.023	0.035	FLT

**Table 12.** Difference between estimated and reference positioning using a VR at various distances for a session length of 15 min, considering the LEIAR10 antenna splitted, and only the GPS constellation.

	**NVS (LEIAR10 Antenna, only GPS, Session Length** **=** **15 min)**						

	**μE [m]**	**μN [m]**	**μh [m]**	**σE [m]**	**σN [m]**	**σh [m]**	
5 km	0.081	0.003	-0.103	0.005	0.002	0.010	FIX
10 km	0.214	0.192	-0.098	0.011	0.012	0.023	FLT
25 km	0.131	0.082	0.112	0.014	0.011	0.034	FLT

**Table 13.** Percentage of DGPS corrections for a session length of about four hours.

**NVS Receiver**	**u-blox Receiver**
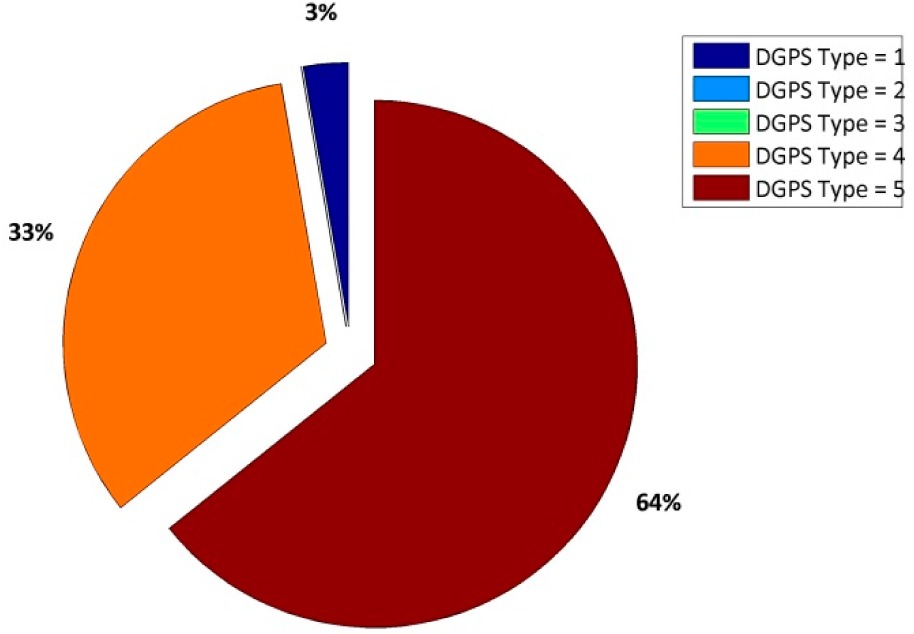	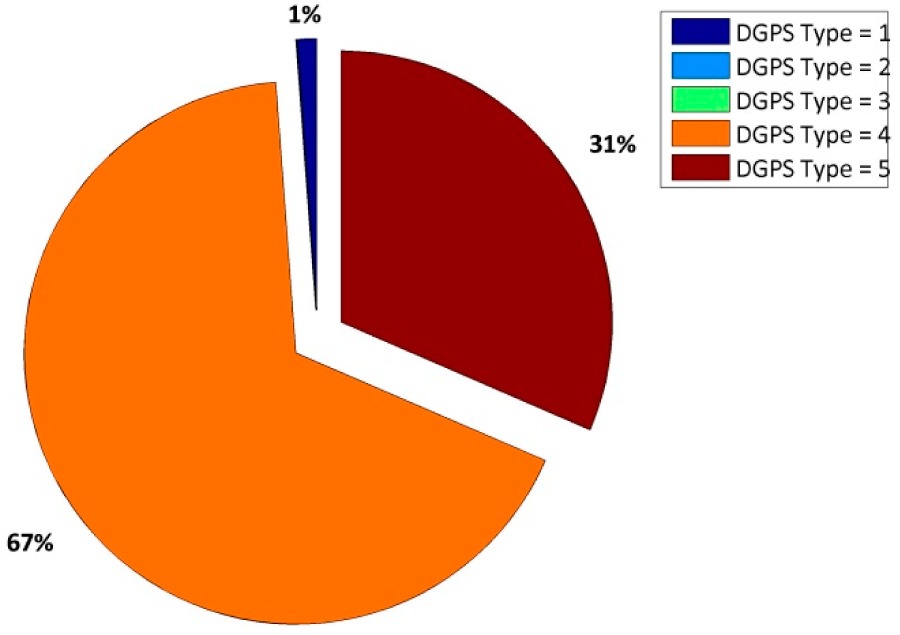

**Table 14.** Planimetric errors obtained with the LEIAR10 antenna and VRS correction.

	**NVS**	**u-blox**
Estimated-reference coordinates		
East [m]	0.003	0.002
North [m]	−0.012	−0.012
h [m]	0.177	0.164
Planimetric residual at 95% [m]	0.017	0.014
Altimetric residual at 95% [m]	0.186	0.168
Percentage of FIX epochs	33%	67.30%

**Table 15.** Planimetric errors considering the Garmin antenna and VRS correction.

	**NVS**	**u-blox**
Estimated-reference coordinates		
East [m]	N/A *	−0.001
North [m]	N/A *	−0.008
h [m]	N/A *	0.234
Planimetric residual at 95% [m]	0.797	0.009
Altimetric residual at 95% [m]	0.504	0.237
Percentage of FIX epochs	0.0%	97.2%

* N/A = no fixed epochs available.

**Table 16.** Planimetric errors considering the LEIAR10 antenna and VRS correction—only GPS constellation.

	**NVS**	**u-blox**
Estimated-reference coordinates		
East [m]	0.107	−0.002
North [m]	0.112	0.006
h [m]	0.192	0.081
Planimetric residual at 95% [m]	0.186	0.009
Altimetric residual at 95% [m]	0.283	0.113
Percentage of FIX epochs	13.6%	95.3%

**Table 17.** Planimetric errors obtained with the Garmin antenna and VRS correction considering only the GPS constellation.

	**NVS**	**u-blox**
Estimated-reference coordinates		
East [m]	−0.231	−0.001
North [m]	−0.123	−0.008
h [m]	0.294	0.234
Planimetric residual at 95% [m]	0.259	0.009
Altimetric residual at 95% [m]	0.352	0.237
Percentage of FIX epochs	7.6%	97.2%

**Table 18.** Difference between estimated and reference positioning considering the LEIAR10 antenna splitted and the GPS + GLONASS constellations.

	**NVS (LEIAR10 Antenna, GPS** **+** **GLONASS)**						

	**μE [m]**	**μN [m]**	**μh [m]**	**σE [m]**	**σN [m]**	**σh [m]**	**%** **of Epochs**
FIX	N/A	N/A	N/A	N/A	N/A	N/A	0%
FLOAT	−0.406	−0.171	0.501	0.052	0.164	0.217	100%

	**u-blox (LEIAR10 Antenna, GPS** **+** **GLONASS)**						

	**μE [m]**	**μN [m]**	**μh [m]**	**σE [m]**	**σN [m]**	**σh [m]**	**%** **of Epochs**

FIX	−0.004	−0.012	0.076	0.035	0.048	0.067	85%
FLOAT	0.131	0.530	1.088	0.023	0.109	0.240	11%

**Table 19.** Difference between estimated and reference positioning considering the LEIAR10 antenna splitted and the only GPS constellation.

	**NVS (LEIAR10 Antenna, only GPS)**						

	**μE [m]**	**μN [m]**	**μh [m]**	**σE [m]**	**σN [m]**	**σh [m]**	**%** **of Epochs**
FIX	−0.094	0.110	0.474	0.124	0.176	0.490	72%
FLOAT	−0.752	−0.166	0.554	0.217	0.125	0.726	25%

	**u-blox (LEIAR10 Antenna, only GPS)**						

	**μE [m]**	**μN [m]**	**μh [m]**	**σE [m]**	**σN [m]**	**σh [m]**	**%** **of Epochs**

FIX	0.008	0.009	0.054	0.051	0.039	0.070	86%
FLOAT	−0.019	−0.219	0.244	0.109	0.414	0.283	9%

**Table 20.** Difference between estimated and reference positioning considering the Garmin antenna splitted and the GPS + GLONASS constellations.

	**NVS (Garmin Antenna, GPS** **+** **GLONASS)**						

	**μE [m]**	**μN [m]**	**μh [m]**	**σE [m]**	**σN [m]**	**σh [m]**	**%** **of epochs**
FIX	N/A	N/A	N/A	N/A	N/A	N/A	0%
FLOAT	−0.594	0.049	0.198	0.064	0.107	0.299	100%

	**u-blox (Garmin Antenna, GPS** **+** **GLONASS)**						

	**μE [m]**	**μN [m]**	**μh [m]**	**σE [m]**	**σN [m]**	**σh [m]**	**%** **of epochs**

FIX	0.020	−0.034	0.052	0.041	0.034	0.069	78%
FLOAT	−0.612	0.182	−0.263	0.045	0.219	0.271	19%

**Table 21.** Difference between estimated and reference positioning considering the Garmin antenna splitted and the only GPS constellation.

	**NVS (Garmin Antenna, only GPS)**						

	**μE [m]**	**μN [m]**	**μh [m]**	**σE [m]**	**σN [m]**	**σh [m]**	**%** **of Epochs**
FIX	0.184	0.225	−0.654	0.798	0.443	0.363	70%
FLOAT	−0.279	0.914	−0.261	2.392	1.238	0.690	25%

	**u-blox (Garmin Antenna, only GPS)**						

	**μE [m]**	**μN [m]**	**μh [m]**	**σE [m]**	**σN [m]**	**σh [m]**	**%** **of Epochs**

FIX	−0.018	0.026	0.048	0.071	0.022	0.058	80%
FLOAT	−0.459	0.593	−0.188	0.176	0.105	0.303	13%
